# Magnetic resonance arthrography with positional manoeuvre for the diagnosis of synovial fold of posterior shoulder joint capsule

**DOI:** 10.1007/s00330-024-10982-3

**Published:** 2024-07-26

**Authors:** Papatya Keles, Hayri Ogul, Kutsi Tuncer, Zakir Sakci, Mutlu Ay, Mecit Kantarci

**Affiliations:** 1 Department of Anatomy, Hamidiye Faculty of Medicine, University of Health Sciences, Istanbul, Turkey; 2 Department of Radiology, Medical Faculty, Medipol University, Istanbul, Turkey; 3 Department of Orthopedic Surgery, Medical Faculty, Altinbas University, Istanbul, Turkey; 4 Department of Radiology, Health Sciences University, Umraniye Training and Research Hospital, Istanbul, Turkey; 5 Department of Radiology, Medical Faculty, Erzincan Binali Yildirim University, Erzincan, Turkey

**Keywords:** Shoulder joint, Joint capsule, Magnetic resonance imaging, Arthrography

## Abstract

**Objectives:**

The objective of this study is to prospectively assess the effectiveness of shoulder magnetic resonance (MR) arthrograms with positional manoeuvres in detecting posterior synovial folds.

**Methods:**

Two radiologists independently assessed all axial MR arthrograms in internal rotation, neutral position, and external rotation for the presence of a posterior synovial fold. The diagnostic performances of the MR arthrograms were then compared, with results validated through arthroscopy.

**Results:**

Arthroscopy was performed on 81 of the 150 patients included in the study. A posterior synovial fold was identified arthroscopically in eleven of these patients. Measurements of the posterior synovial fold obtained in external rotation and the neutral position of the arm showed a significant correlation with arthroscopic results (*p* < 0.05). For detecting the posterior synovial fold with arthroscopic correlation, the sensitivity and specificity values for observer 1 and observer 2 were 100–81.4% and 100–88.6%, respectively, for MR arthrograms in the neutral position; 100–52.9% and 100–62.9% for MR arthrograms in external rotation; and 100–95.7% and 81.8–98.6% for MR arthrograms in internal rotation. There was a fair agreement for MR arthrography in external rotation for detecting posterior synovial folds, while MR arthrograms in internal rotation and neutral position showed near-perfect and significant interobserver agreement.

**Conclusion:**

The rotational positions of the humeral neck during MR arthrographic examination can influence the diagnostic specificity and sensitivity of axial MR arthrograms in detecting the posterior synovial fold.

**Clinical relevance statement:**

The posterior synovial fold can mimic a posterior labral detachment. Therefore, its correct identification is crucial in order to avoid unnecessary surgical procedures.

**Key Points:**

- *Movement of the shoulder may introduce variability in MR arthrography appearance*.
- *Rotation of the humeral neck during MR arthrography can affect diagnoses in posterior synovial fold detection*.
- *Given that posterior synovial folds can imitate posterior labral detachment, their correct identification is crucial to avoid unnecessary surgical procedures*.

## Introduction

Posterior labrocapsular pathologies of the shoulder joint are relatively uncommon. Main indications for posterior shoulder joint magnetic resonance (MR) arthrography include reverse fibrous or bony Bankart lesions, superior labrum anterior posterior (SLAP) lesions with posterior extension, posterior labrocapsular periosteal sleeve avulsion lesions, Kim’s lesions, Bennett lesions, and reverse glenolabral articular disruption lesions [1–3]. Posterior instability, which accounts for only 2–4% of all glenohumeral instabilities, results in a reverse Bankart lesion [1, 2]. Many MR imaging and arthroscopy studies have identified abnormalities in the labral insertion to the posterior glenoid margin and morphological variations of the glenoid labrum. These anatomical variations, such as notched and cleaved shapes of the glenoid labrum and sublabral cleft or loose attachment of the labrum to the glenoid bone, can mimic posterior labral lesions like labral defects and detachments. Therefore, careful interpretation of shoulder MR imaging and MR arthrography slices is essential for correctly diagnosing posterior labrocapsular lesions and variations [3]. Another variation of the posterior shoulder joint structure that can mimic labral detachment is a synovial fold anomaly [4]. Synovial fold variations in the posterior capsule of the shoulder joint are localised thickenings of the joint capsule, similar to the glenohumeral ligaments in the anterior and inferior capsule [5, 6]. Like the glenohumeral ligaments, this anatomical variation may contribute to the passive stabilisation of the shoulder joint, although no biomechanical studies have confirmed this. Although no radiologic studies show the posterior synovial fold is symptomatic, it is believed these ligament-like capsular thickenings may cause internal impingement, similar to plicae in the knee and elbow. In addition, the presence of many pathologies accompanying the posterior synovial fold in the shoulder joint may obscure the clinical manifestations of this anatomical variation. The synovial fold of the posterior shoulder joint capsule is a rare anatomical variation well-defined on MR arthrograms [5, 6]. Accurate identification of this rare capsular anomaly is crucial to avoid unnecessary surgical procedures.

Although conventional MR imaging is commonly used to evaluate the posterior labrocapsular structures of the shoulder joint, MR arthrography with intra-articular gadolinium injection is a more effective imaging technique for detecting posterior labrocapsular anatomy, variations, and pathologies [3, 5, 7, 8]. On shoulder MR arthrography, intra-articular anatomic structures such as the glenoid labrum, glenohumeral ligaments, and joint capsule can be distinctly identified due to adequate capsular distention and the intra-articular contrast effect [7, 9–11]. MR arthrography of the shoulder without stress manoeuvres has previously been used as an imaging modality for detecting the posterior capsular fold [5, 6]. However, the additional value of MR arthrography with the arm positioned in internal and external rotation has not been comprehensively investigated. We hypothesise that shoulder MR arthrography in internal and external rotation would provide additional value for detecting posterior synovial folds. Therefore, in this study, we aimed to compare the rate and discrimination ability of MR arthrography to detect posterior synovial folds in internal rotation, external rotation, and neutral position separately and to correlate the findings with arthroscopic surgery, the gold standard.

## Methods and materials

### Patients

**Fig. 1:**
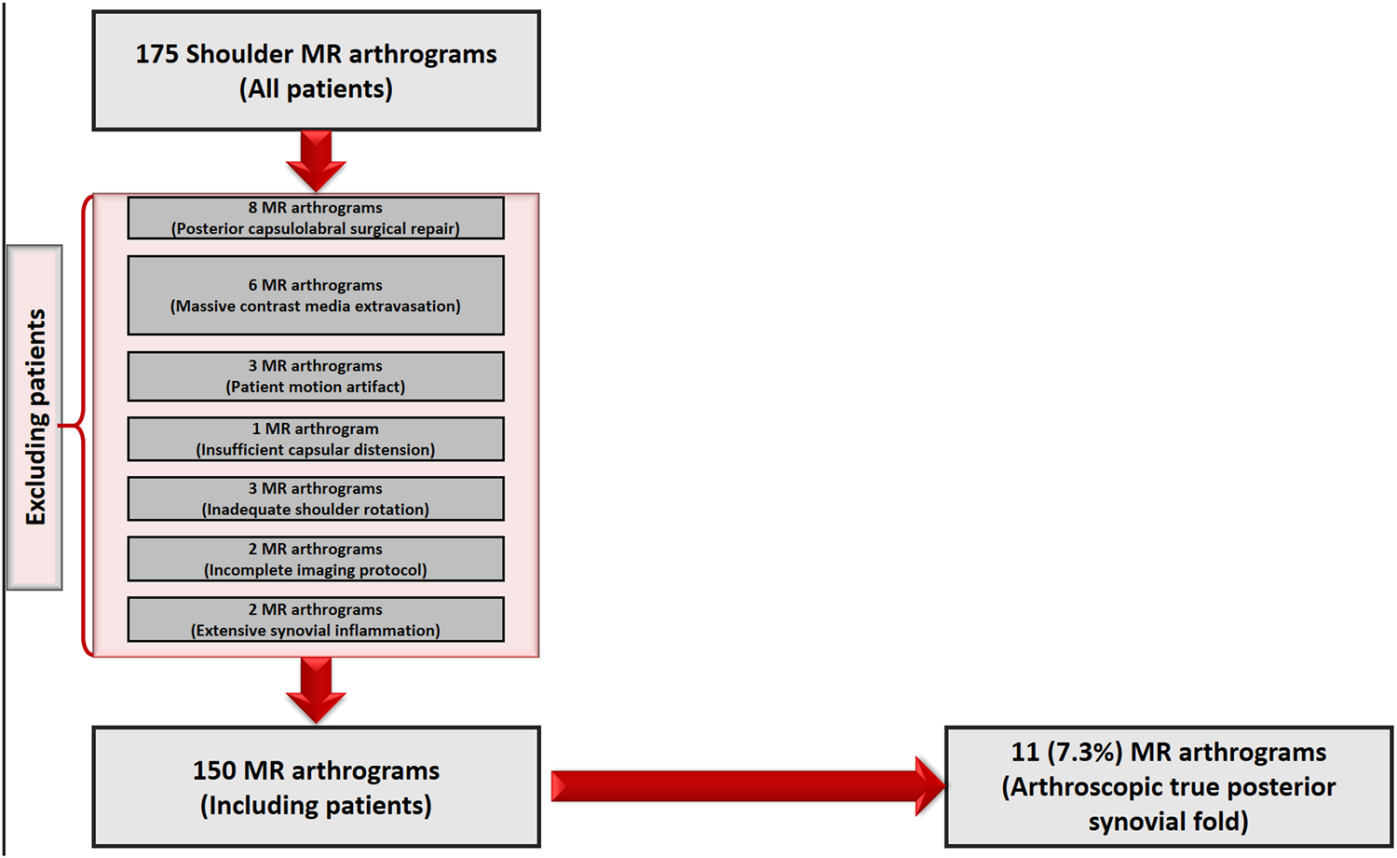
A flowchart shows exclusion criteria and patient selection

A total of 175 consecutive MR arthrography images from subjects referred to the radiology department for shoulder joint MR arthrography between March 2019 and January 2022 were prospectively reviewed for the presence of the posterior synovial fold. The main indications for MR arthrography included anterior labrum tears, SLAP lesions, posterior labral injuries, rotator cuff tendon tears, and biceps tendon pathologies. Exclusion criteria comprised a history of posterior labrocapsular surgery, inadequate capsular extension, massive posterior pericapsular contrast media extravasation, patient motion artefacts, inadequate shoulder rotation, diffuse synovial inflammation, and incomplete MR arthrography protocols. Twenty-five of the 175 patients were excluded from the study. A patient flowchart detailing the exclusion criteria and patient selection is provided (Fig. 1). The institutional review board approved the study (2019/302), and written informed consent was obtained from all patients for the arthrographic procedure.

### Glenohumeral joint arthrography technique and MR arthrography imaging procedure

**Fig. 2:**
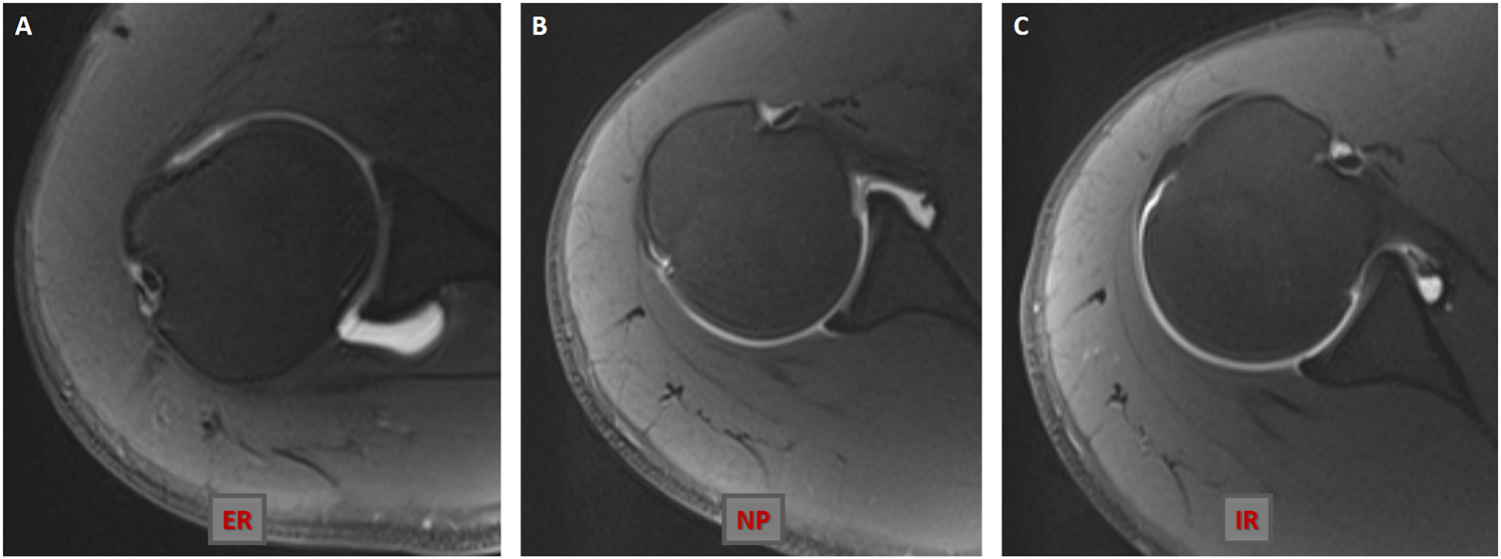
Axial fat-suppressed turbo spin echo (TSE) T1-weighted MR arthrograms (**A**–**C**) obtained at the same level show a normal arthrographic anatomy of the posterior shoulder joint capsule in the external rotation, neutral position, and internal rotation of the ipsilateral arm. ER, external rotation; IR, internal rotation; NP, neutral position

For MR arthrography, a gadolinium-based solution (0.1 mL gadopentetate dimeglumine (Magnevist; Bayer Schering Pharma, Germany) diluted with 20 mL saline) at a ratio of 1:200 was injected by two radiologists through a posterior approach under ultrasound guidance. All injections were performed with a 20-gauge needle using the Applio ultrasound system (Toshiba Medical Systems, Tokyo, Japan). Adequate joint distension was achieved with an injection volume of 10 to 20 mL. All MR arthrographic examinations were conducted using a 3-T MR device (Magnetom Skyra; Siemens Healthcare, Erlangen, Germany) with a superficial coil, 15–30 min after joint injection. Our routine shoulder MR arthrography protocol includes fat-suppressed turbo spin echo T1-weighted (transverse, oblique sagittal, and oblique coronal planes), 3D volumetric T1-weighted (often fat-suppressed volumetric interpolated breath-hold examination), and fluid-sensitive T2-weighted (often oblique coronal half Fourier single-shot turbo spin-echo) MR arthrography sequences. This protocol is summarised in Table 1. In addition, for this study, we obtained transverse fat-suppressed T1-weighted shoulder MR arthrograms with the arm in internal and external rotation and in the neutral position (Fig. 2).

**Table 1:** 3-Tesla MR scanner routine MR arthrography sequence parameters

Sequence	TR/TE (ms)	ETL	Plane	Flip angle (degrees)	Fat-suppression	Slice thickness (mm)	Intersection gap (mm)	Matrix size	FOV (mm)
TSE T1	565/10	8	Oblique sagittal, oblique coronal, and transvers	−	−, +, +	3	3.5	256 × 256	160
TSE T2	4800/70	8	Oblique coronal	−	−	1	1.2	320 × 192	160
3-D T1 VIBE	8/3.5	−	Oblique coronal	11	+	0.6	0	512 × 512	160

### Image analysis

All MR arthrograms were reviewed on a high-resolution monitor of a picture archiving and communication system (Sectra AB, Linköping, Sweden). To compare the diagnostic performance of MR arthrograms in different arm rotations, axial plane MR arthrography images obtained in internal rotation, neutral position, and external rotation were independently and separately evaluated for the presence of a posterior synovial fold by two radiologists—one with 17 years of musculoskeletal radiology experience and the other with 6 years of experience. Both radiologists were blinded to the patients’ histories and previous imaging findings. Each radiologist independently assessed the axial MR arthrograms in each arm position and recorded their findings.

The technique developed by Ogul et al was used to measure the synovial fold in axial MR arthrograms [5]. Focal capsular plications of less than 2 mm in the posterior joint capsule were included in the measurements to fully reflect positional changes in the posterior shoulder joint capsule but were not considered as posterior synovial fold variations. Focal thickening of 2 mm or more in the craniocaudal oblique direction in the posterior shoulder joint capsule detected on MR arthrography images was classified as a synovial fold [5, 6]. In the final stage, both radiologists reviewed all MR arthrograms together, and the dimensions of the posterior synovial fold were measured by consensus. An interobserver evaluation was then performed for all arthrograms.

### Arthroscopic correlation

The time interval between the MR arthrography interpretation and the arthroscopy procedure ranged from two weeks to two months. A significant portion of the patients had glenoid labrum and rotator cuff tendon tears. Of the 150 patients, 81 (54%) underwent arthroscopic treatment by a shoulder orthopaedic surgeon to repair the labrum or rotator cuff tendon injuries. These patients were also examined for posterior synovial fold abnormalities. Sensitivity and specificity assessments were performed for the MR results of these 81 patients, correlated with arthroscopy findings.

### Statistical analysis

All statistical analyses were conducted using the SPSS software programme (version 25). The assumption of normality was assessed using the Kolmogorov–Smirnov and Shapiro–Wilk tests. Homogeneity assumption of group variances was evaluated using the Levene test. For intergroup comparisons, continuous variables with a normal distribution were analysed using independent samples *t*-tests, while those without a normal distribution were assessed using the Mann–Whitney *U* and Kruskal–Wallis tests. Categorical ratios were compared using a Chi-square test. Descriptive analyses were presented as median (minimum–maximum) and mean ± standard deviation (SD). Sensitivity and specificity values were calculated for each rotational MR arthrography image (external rotation, neutral position, and internal rotation) in detecting a synovial fold of the posterior shoulder joint capsule. Interobserver agreement for MR arthrography in the arm’s neutral position and MR arthrographic images with internal and external positions was determined using the kappa coefficient method proposed by Landis and Koch (0–0.20: slight agreement; 0.21–0.40: fair agreement; 0.41–0.60: moderate agreement; 0.61–0.80: substantial agreement; 0.81–1.00: near-perfect agreement). A *p*-value of ≤ 0.05 was considered statistically significant.

## Results

Examiners evaluated 175 MR arthrograms from 175 consecutive patients, all with unilateral shoulder imaging. Twenty-five MR arthrograms were excluded from the study. Among the 150 patients whose shoulder joints were examined, axial T1-weighted MR arthrograms were obtained with the arm in external rotation, neutral position, and internal rotation. Arthroscopy, used as the gold standard, was performed in 81 (54%) of the 150 shoulder joints. Demographic data are summarised in Table 2.

**Table 2:** Demographic data of the patients

	Male (*n* = 100)	Female (*n* = 50)	*p*	Total (*n* = 150)
Age (years), mean ± SD	40 ± 13 (18–76)	37 ± 14 (18–63)	0.71	38 ± 14 (18–76)
Side, *n* (%)				
Right	52 (52)	34 (68)	0.30	86 (57.3)
Left	48 (48)	16 (32)		64 (42.7)

*SD* standard deviation

According to the consensus synovial fold measurements, most arthrographic synovial fold anomalies were diagnosed in the external rotation position. Synovial fold measurement values are presented in Table 3. No significant difference was found among patients regarding the affected shoulder side, gender, and the prevalence of synovial fold (*p* > 0.05).

**Table 3:** Synovial fold measurement revealed by consensus

	*N*	Minimum (millimeter)	Maximum (millimeter)	Mean (millimeter)	Std. Deviation
Neutral position	26	0.5	9.0	2.569	1.5255
External rotation	55	2.0	9.0	2.967	1.1172
Internal rotation	14	1.3	9.0	2.686	1.8765

The incidence of shoulder pathologies on arthroscopy did not significantly differ with the presence of posterior synovial fold (*p* > 0.05). The most commonly associated pathologies in patients diagnosed with posterior synovial fold arthroscopically were SLAP lesions and rotator cuff tendon ruptures (Table 4). Measurement values of posterior synovial plications performed in external rotation and the neutral position of the arm showed a significant correlation with arthroscopic results (*p* < 0.05) (Table 5). Most of the posterior synovial plications detected in external rotation were indicative of likely pseudo folds.

**Table 4:** Relationship between arthroscopic results and demographic variables

	Absence of posterior synovial fold *n* (%)	Presence of posterior synovial fold *n* (%)	Total *n* (%)	Chi-square	*p*
Patient gender					
Male	45 (83.3)	9 (16.7)	54 (100)	1.315	0.252
Female	25 (92.6)	2 (7.4)	27 (100)		
Shoulder side					
Right	38 (88.4)	5 (11.6)	43 (100)	0.298	0.585
Left	32 (84.2)	6 (15.8)	38 (100)		
Associated pathology					
None	3 (75)	1 (25)	4 (100)	6.273	0.307
Bankart or Bankart variant	17 (89.5)	2 (10.5)	19 (100)		
Revers Bankart or variant	2 (100)	0 (0)	2 (100)		
SLAP lesion	36 (90)	4 (10)	40 (100)		
Rotator cuff tendon partial rupture	4 (57.1)	3 (42.9)	7 (100)		
Rotator cuff tendon full-thickness rupture	6 (85.7)	1 (14.3)	7 (100)		
Other pathologies	2 (100)	0 (0)	2 (100)

**Table 5:** Relationship between arthroscopic results and measurement values of posterior synovial plication in internal rotation, external rotation, and neutral position

	MR arthrograpy measurement results of patients without posterior synovial fold during arthroscopy $\bar{\boldsymbol x}\pm {\mathbf {SS}}\,/$ Med (min-max)	MR arthrography measurement results of patients with posterior synovial fold during arthroscopy $\bar{\boldsymbol x}\pm {\mathbf {SS}}\,/$ Med (min-max)	Total $\bar{\boldsymbol x}\pm {\mathbf {SS}}\,/$ Med (min-max)	U/t	*p*
	37.59 ± 13.43	42 ± 12.85	38.19 ± 13.36	−1.019^t^	0.311
Neutral position	2.1 (0.5–4)	2.6 (2.1–9)	2.5 (0.5–9)	−2.211^U^	0.026*
Internal rotation	2.8 (2–4.7)	3.7 (2.4–9)	2.9 (2–9)	−2.785^U^	0.005*
External rotation	1.6 (1.3–3)	2.2 (2–9)	2.15 (1.3–9)	−1.019^U^	0.368

* *p* < 0,05 | ^U^: Mann–Whitney *U* test value | ^t^: Independent sample *t-*test calculation value

**Fig. 3:**
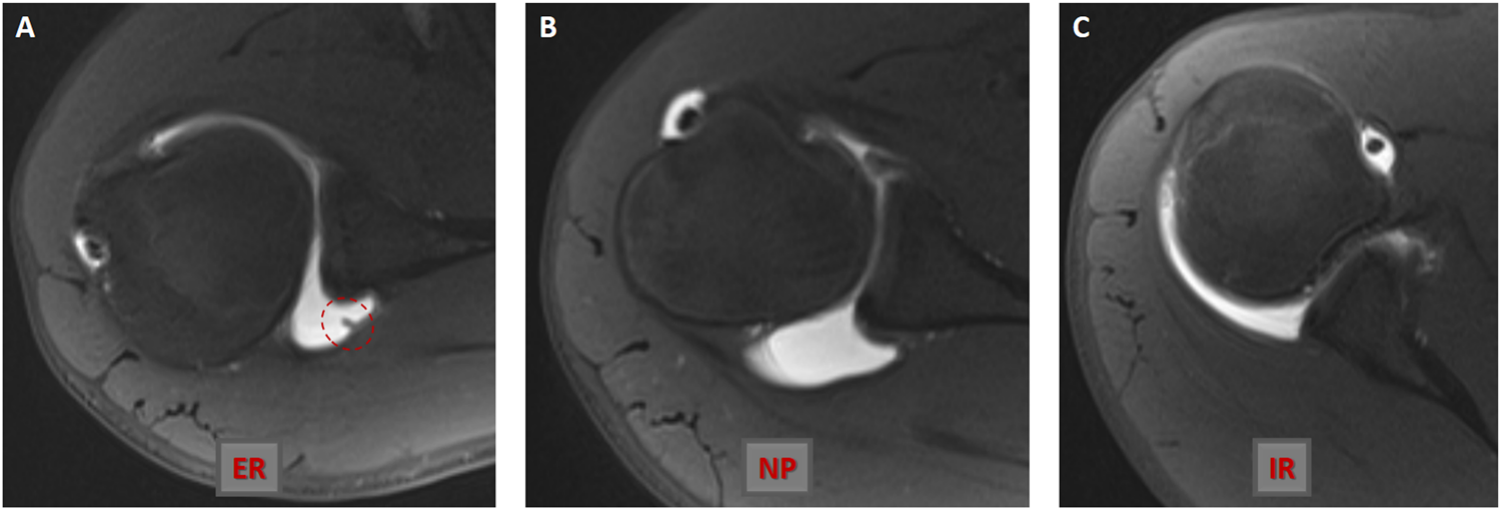
Axial fat-suppressed TSE T1-weighted MR arthrogram (**A**) obtained in the external rotation of the ipsilateral arm shows a synovial plication (circle) in the posterior shoulder joint capsule. Axial fat-suppressed TSE T1-weighted MR arthrography sections (**B**, **C**) passing through the same level as in Fig. 1A show normal anatomy of the posterior shoulder joint capsule in the neutral position and internal rotation of the ipsilateral arm. ER, external rotation; IR, internal rotation; NP, neutral position

**Fig. 4:**
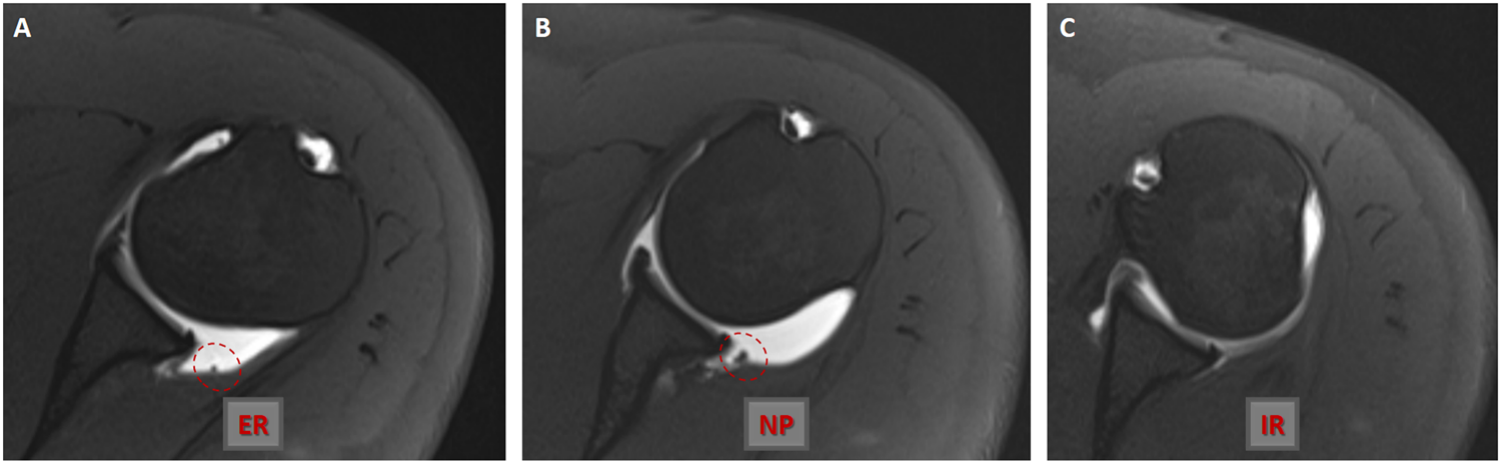
Axial fat-suppressed TSE T1-weighted MR arthrograms (**A** and **B**) obtained at the same level in the external rotation and neutral position of the ipsilateral arm show a synovial plica (circle) in the posterior shoulder joint capsule. There is no synovial fold in axial fat-suppressed TSE T1-weighted MR arthrogram (**C**) obtained at the same level in the internal rotation of the ipsilateral arm. ER, external rotation; IR, internal rotation; NP, neutral position

**Fig. 5:**
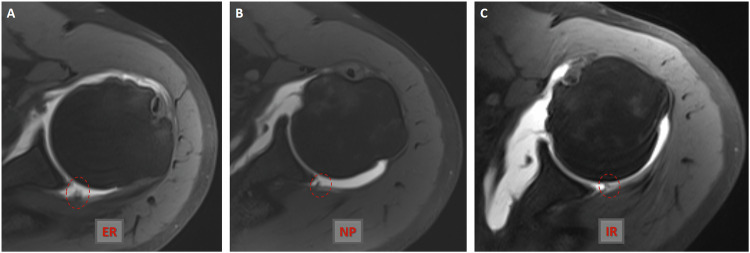
Axial fat-suppressed TSE T1-weighted MR arthrograms (**A**–**C**) obtained at the same level show a true synovial fold anomaly (circle) of the posterior shoulder joint capsule in the external rotation, neutral position, and internal rotation of the ipsilateral arm. ER, external rotation; IR, internal rotation; NP, neutral position

All MR arthrograms, including those in neutral position and internal/external rotation, demonstrated high sensitivity in diagnosing posterior synovial fold. MR arthrograms evaluated with the arm in internal rotation showed high sensitivity, while those evaluated in external rotation had lower sensitivity. For posterior synovial fold detection correlated with arthroscopy, sensitivity and specificity values for observer 1 and observer 2 were as follows: 100–81.4%/100–88.6% for MR arthrograms in the neutral position, 100–52.9%/100–62.9% for MR arthrograms in external rotation, and 100–95.7%/81.8–98.6% for MR arthrograms in internal rotation, respectively (Supplementary Table 1). In terms of interobserver variability assessment, MR arthrography in external rotation demonstrated moderate agreement for detecting posterior synovial folds (Fig. 3), while MR arthrograms in internal rotation and neutral position showed near-perfect and significant interobserver agreement, respectively (Figs. 4 and 5; Supplementary Table 1).

## Discussion

Elaborate investigations of the location and type of the posterior synovial fold on shoulder MR imaging and MR arthrography have been reported in only a few studies [4–6]. In these studies, all MR examinations were obtained in the neutral position of the arm. Unlike these, in our study, MR arthrograms were obtained with the arm in a neutral position, external rotation, and internal rotation, and MR arthrograms in internal and external rotation were prospectively compared with the neutral position value for the posterior synovial folds. Our results, using arthroscopy as the reference standard, demonstrated that MR arthrograms of the shoulder obtained with the arm in internal rotation had higher specificity and relatively lower sensitivity for detecting posterior synovial folds compared to MR arthrograms in neutral position and external rotation, with inter-examiner agreement close to perfect. To the best of our knowledge, there is no MR arthrography study in the literature investigating the posterior synovial folds of the shoulder joint with the arm in external and internal rotation.

The synovial fold or plica, which can be considered as a remnant of embryonic septa during normal joint development, is a focal thickened fibrotic tissue extending from the joint capsule. Synovial folds are most common in the knee and elbow joints and are usually non-functional and asymptomatic. However, if synovial folds become thickened and inflamed, they may cause clinical complaints [12].

Cases with symptoms are termed synovial fold syndrome or soft tissue impingement syndrome and, in such instances, may mimic other joint pathologies. Synovial fold anomaly is considerably less common in the shoulder joint compared to other joints, with shoulder synovial fold abnormalities typically described as localised posterior and superior to the joint capsule. While there are several reported symptomatic cases of superior synovial folds in the shoulder joint, there are no documented symptomatic cases of posterior synovial folds [13]. In a study correlating shoulder imaging with arthroscopy, Novak et al noted a prevalence of posterior synovial folds of 2.7% and 1.5% on MR imaging and MR arthrography, respectively [6]. More recently, Ogul et al reported a prevalence of 5.8% for posterior synovial folds in the shoulder joint based on MR arthrography [5]. However, our study revealed a notably higher prevalence of posterior synovial folds (16.5%) in the shoulder joint. Several factors could explain this disparity: firstly, our study had a prospective design, optimising all MR sequence parameters, while the previous studies were retrospective. In addition, our arthroscopist specifically examined the posterior joint capsule for synovial fold abnormalities. Secondly, cumulative experience with posterior capsular abnormalities may have facilitated the diagnosis of posterior synovial folds during both arthrography and arthroscopy. Lastly, differences in patient populations may account for the higher prevalence observed in our study.

MR arthrographic examination of the shoulder utilising positional manoeuvres, such as internal/external rotation, abduction and external rotation, and adduction internal rotation positions of the humeral head, has been reported in previous literature. These studies have demonstrated the high utility of shoulder MR arthrograms evaluated using positional manoeuvres in assessing the shoulder’s labroligamentous lesions [14–19]. However, most studies have focused on the anterior and superior capsulolabroligamentous complex. There is a notable lack of MR arthrographic examination of the posterior labrocapsular structures using positional manoeuvres. The posterior joint capsule is relatively weaker than the anterior capsular structure due to the absence of glenohumeral ligaments in the posterior aspect of the shoulder joint. Furthermore, posterior labrocapsular structures tend to be less affected than anterior structures. While not yet confirmed, the synovial fold of the posterior shoulder capsule may contribute to the passive stability of the posterior capsule. In addition, this abnormal capsular thickening can mimic posterior labral lesions in traditional MR images [4, 5]. Therefore, accurate diagnosis is crucial to avoid unnecessary surgical procedures. Shoulder MR arthrography outperforms non-arthrographic MR imaging in diagnosing posterior synovial folds and demonstrates high diagnostic specificity and sensitivity [5].

MR arthrographic examination typically involves a supine patient with the arm positioned in either the neutral position or a slight external rotation. Excessive external rotation can lead to laxity in the posterior joint capsule, while internal rotation creates capsular tension. Through our routine reporting of shoulder MR imaging and MR arthrograms, we observed that a loose posterior capsule can sometimes mimic a pseudo-fold, whereas a tight shoulder capsule may obscure the true synovial fold. Thus, this study aimed to investigate MR arthrographic examination of the shoulder using positional manoeuvres, such as neutral position, internal rotation, and external rotation, to detect posterior synovial folds.

Our findings revealed that axial MR arthrograms obtained with external rotation of the humeral head depicted a higher incidence of posterior synovial folds compared to those obtained with internal rotation or in the neutral position. This suggests that MR arthrography in external rotation exhibits a lower specificity value for diagnosing a posterior synovial fold (Observer 1 = 52.9% and Observer 2 = 62.9%). However, nearly all MR arthrograms in our patient cohort, regardless of stress manoeuvres, demonstrated high sensitivity in detecting the synovial fold. Among these, MR arthrographic examination of the shoulder using internal rotation exhibited the highest sensitivity (Observer 1 = 65.7% and Observer 2 = 98.6%).

Our study is subject to several limitations: First, although arthroscopy served as the reference standard for diagnosing the posterior synovial fold, only a subset of patients could be correlated with arthroscopy. Second, none of our patients underwent histopathologic or biomechanical correlations. Therefore, future cadaveric studies may provide insights into the histologic features and mechanical implications of the posterior synovial fold.

## Conclusion

This study observed that the axial MR arthrographic presentation of the posterior synovial fold varies with internal or external rotation of the arm. Regardless of positional manoeuvring, all MR arthrograms exhibited high sensitivity in detecting the posterior synovial fold. However, the diagnostic specificity of identifying the posterior synovial fold significantly improves with axial MR arthrography in internal rotation compared to MR arthrography of the shoulder in the neutral position or external rotation.

## Supplementary information

ELECTRONIC SUPPLEMENTARY MATERIAL

## Abbreviations

MR: Magnetic resonance
SLAP: Superior labrum anterior posterior
