# Supplementary material for: Magnetic resonance arthrography with positional manoeuvre for the diagnosis of synovial fold of posterior shoulder joint capsule
Source: Eur Radiol. 2024 Jul 26;35(2):848–55. doi: 10.1007/s00330-024-10982-3 (PMC11782312; doi:10.1007/s00330-024-10982-3)
Supplement: Supplementary file 1 — ELECTRONIC SUPPLEMENTARY MATERIAL [file 330_2024_10982_MOESM1_ESM.pdf]

Magnetic Resonance Arthrography with Positional Manoeuvre for the Diagnosis of Synovial Fold of Posterior Shoulder Joint Capsule

ELECTRONIC SUPPLEMENTARY MATERIAL

Supplementary Table 1: Comparison values between the arthroscopic results and the MR arthrography findings of both observers according to posterior synovial plications.

	Arthroscopy						
	Absence of posterior synovial fold n (%)	Present of posterior synovial fold n (%)	Total n (%)	Kappa	p	Sensitivity	Specificity
**MR arthrography**							
**Posterior synovial plica on internal rotation (Observer 1)**							
**Absence of posterior synovial plica**	67 (95,7)	0 (0)	67 (82,7)	0,858	<0,001*	%100	%95,7
**Presence of posterior synovial plica**	3 (4,3)	11 (100)	14 (17,3)				
**Posterior synovial plica on internal rotation (Observer 2)**							
**Absence of posterior synovial plica**	69 (98,6)	2 (18,2)	71 (87,7)	0,836	<0,001*	%81,8	%98,6
**Presence of posterior synovial plica**	1 (1,4)	9 (81,8)	10 (12,3)				
**Posterior synovial plica on neutral position (Observer 1)**							
**Absence of posterior synovial plica**	57 (81,4)	0 (0)	57 (70,4)	0,544	<0,001*	%100	%81,4
**Presence of posterior synovial plica**	13 (18,6)	11 (100)	24 (29,6)				
**Posterior synovial plica on neutral position (Observer 2)**							
**Absence of posterior synovial plica**	62 (88,6)	0 (0)	62 (76,5)	0,678	<0,001*	%100	%88,6
**Presence of posterior synovial plica**	8 (11,4)	11 (100)	19 (23,5)				
**Posterior synovial plica on external rotation (Observer 1)**							
**Absence of posterior synovial plica**	37 (52,9)	0 (0)	37 (45,7)	0,233	0,001*	%100	%52,9
**Presence of posterior synovial plica**	33 (47,1)	11 (100)	44 (54,3)				
**Posterior synovial plica on external rotation (Observer 2)**							
**Absence of posterior synovial plica**	44 (62,9)	0 (0)	44 (54,3)	**0,315**	<0,001*	%100	%62,9
**Presence of posterior synovial plica**	26 (37,1)	11 (100)	37 (45,7)				
